# Recent Developments and Strategies for the Application of *Agrobacterium*-Mediated Transformation of Apple *Malus* × *domestica* Borkh

**DOI:** 10.3389/fpls.2022.928292

**Published:** 2022-06-30

**Authors:** Susan Schröpfer, Janne Lempe, Ofere Francis Emeriewen, Henryk Flachowsky

**Affiliations:** Julius Kühn Institute (JKI) - Federal Research Centre for Cultivated Plants, Institute for Breeding Research on Fruit Crops, Dresden, Germany

**Keywords:** apple, *Malus*, *Agrobacterium tumefaciens*, transformation, genome editing, virus-induced gene silencing, rapid cycle breeding

## Abstract

Genetic transformation has become an important tool in plant genome research over the last three decades. This applies not only to model plants such as *Arabidopsis thaliana* but also increasingly to cultivated plants, where the establishment of transformation methods could still pose many problems. One of such plants is the apple (*Malus* spp.), the most important fruit of the temperate climate zone. Although the genetic transformation of apple using *Agrobacterium tumefaciens* has been possible since 1989, only a few research groups worldwide have successfully applied this technology, and efficiency remains poor. Nevertheless, there have been some developments, especially in recent years, which allowed for the expansion of the toolbox of breeders and breeding researchers. This review article attempts to summarize recent developments in the *Agrobacterium*-mediated transformation strategies of apple. In addition to the use of different tissues and media for transformation, agroinfiltration, as well as pre-transformation with a Baby boom transcription factor are notable successes that have improved transformation efficiency in apple. Further, we highlight targeted gene silencing applications. Besides the classical strategies of RNAi-based silencing by stable transformation with hairpin gene constructs, optimized protocols for virus-induced gene silencing (VIGS) and artificial micro RNAs (amiRNAs) have emerged as powerful technologies for silencing genes of interest. Success has also been achieved in establishing methods for targeted genome editing (GE). For example, it was recently possible for the first time to generate a homohistont GE line into which a biallelic mutation was specifically inserted in a target gene. In addition to these methods, which are primarily aimed at increasing transformation efficiency, improving the precision of genetic modification and reducing the time required, methods are also discussed in which genetically modified plants are used for breeding purposes. In particular, the current state of the rapid crop cycle breeding system and its applications will be presented.

## Introduction

**Figure 1:**
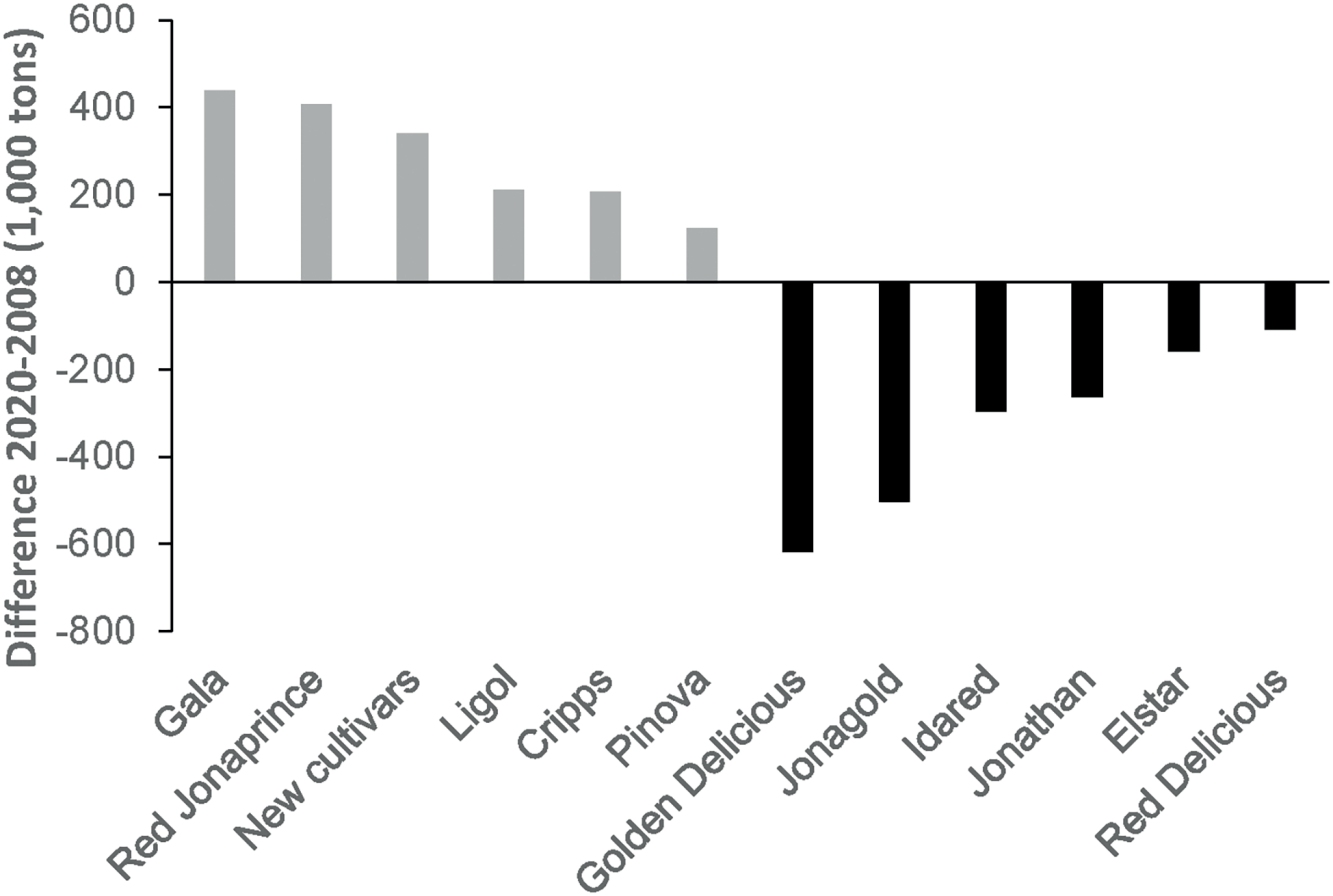
Development of the harvest volumes of selected apple cultivars in the EU in comparison with the years 2008 and 2020 (www.prognosfruit.eu).

The domesticated apple *Malus* ×*domestica* Borkh. is the most important fruit in the temperate climate zone in economic terms. The global gross production value in 2018 was around 51 billion US dollars.^1^ Apples are produced in 93 countries around the world, with about two-thirds of the total quantity produced in China, the United States, Turkey, Italy, and India. Although there are more than 7,500 named apple varieties worldwide (Volk et al., 2015), world apple production has been dominated by only a few top cultivars (e.g., “Delicious,” “Golden Delicious,” “Granny Smith,” “Fuji” and “Gala”) in recent decades (O'rourke, 2003; Hancock et al., 2008). For some years now, however, there has been an increasing change in cultivars on the global market. New cultivars are becoming increasingly important. This becomes very clear when comparing the harvest volumes of the most important apple cultivars in the European Union (EU) between the years 2008 and 2020 (Figure 1). Traditional cultivars such as “Golden Delicious,” “Jonagold” and “Idared” are increasingly being replaced in cultivation by cultivars such as “Gala” (including sports), “Red Jonaprince,” “Ligol,” “Cripps Pink” and “Pinova.”^2^ Of particular interest is a steadily growing segment in which a large number of new cultivars find their place (Figure 1).

These new cultivars are the result of cross-breeding and selection programs in which the most modern breeding methods are increasingly being used.

More than 100 breeding programs worldwide are working on the development of new apple cultivars (Letschka et al., 2021). They are all increasingly benefiting from the developments of the last three decades in the field of breeding research, where great progress has been made in the field of structural genome analysis (Hanke et al., 2020). New sequencing technologies and modern molecular marker systems have facilitated the generation of genetic maps and QTL mapping. Today, more than 120 genetic apple linkage maps are already listed in the Genome Database for Rosaceae.^3^ In addition, first genome sequences of economically important apple cultivars and selected wild species accessions enable simple and rapid identification of candidate genes and genomic regions of interest, as well as the development of robust linked markers suitable for marker-assisted selection. All these advances in breeding research have the potential to significantly accelerate the development of new apple cultivars (Van Nocker and Gardiner, 2014).

Progress has also been made in the field of functional genome analysis. The rapid development in the field of omics technologies (e.g., transcriptomics, proteomics, and metabolomics) has led to a much faster and more targeted cloning of candidate genes (Hanke et al., 2020). However, the functional validation of candidate genes of interest is still difficult and time-consuming. Mutant stocks of apple, as they are made available for *Arabidopsis thaliana via* the TAIR database (Garcia-Hernandez et al., 2002) do not exist and cannot be produced in this form due to the prevailing self-incompatibility and the lack of opportunity to preserve interesting genotypes in the form of seeds. This rules out the simple and rapid phenotypic evaluation of mutants as well as the complementation of the knock-out mutant as possible strategies. A doubtless elucidation of the function of a gene in apple is usually always accompanied by the production of a new genetically modified (gm) plant. Although there are several possibilities for the production of gm plants, genetic transformation using *Agrobacterium tumefaciens* is still the method of choice.

David James and co-workers first described the method of *A. tumefaciens*-mediated leaf disk transformation of apple in 1989 (James et al., 1989). In the following years, most studies focused on improving the methodology to increase transformation efficiency (Hanke et al., 2020). Above all, it was necessary to establish suitable protocols for *in vitro* culture, because of the recalcitrant nature of apple in tissue culture. Nevertheless, over the years it has been possible to develop suitable protocols for different apple genotypes. Teixeira Da Silva et al. (2019) recently summarized the advances and applications in this field. Despite numerous efforts over the last three decades, the genetic transformation of apple is still very difficult. Established protocols are time-consuming and the efficiency of transformation is comparatively low (Hanke et al., 2020). Several publications summarized the efforts and achievements of genetic engineering in apple (Bulley et al., 2007; Gessler and Patocchi, 2007; Bhatti and Jha, 2010; Hanke and Flachowsky, 2010; Rai and Shekhawat, 2014). A lot of work, energy, and effort has gone into developing selection methods as an alternative to *nptII* (Szankowski et al., 2003; Degenhardt et al., 2006) and establishing strategies for developing marker gene-free plants (Krens et al., 2004; Herzog et al., 2012). Although it has been possible to develop the methodology from classical transgenic plants to intragenic (Joshi et al., 2011) and cisgenic plants (Vanblaere et al., 2011; Kost et al., 2015), this way of accelerating the breeding process although successful still lacks acceptance by many consumers, at least in Europe and many other countries around the world. However, at least in the field of functional genome analysis, *Agrobacterium*-mediated transformation has become an established tool.

On this account, work on improving transformation efficiency has also been ongoing in recent years. Among other strategies tested, the agroinfiltration method (Chevreau et al., 2019) and the pre-transformation with the *BABY BOOM* (*BBM*) transcription factor (Chen et al., 2022) appear particularly promising. Significant progress has also been made in establishing methods for the transient expression of gene constructs in apple. Optimized protocols for virus-induced gene silencing (VIGS), virus-induced overexpression of genes (VOX), and silencing mediated by artificial micro RNAs (amiRNAs) have emerged as powerful technologies (Yamagishi et al., 2011; Zhang et al., 2016; Charrier et al., 2019b; Werner Ribeiro et al., 2020). In addition, several research groups have succeeded in establishing methods for targeted genome editing in apple (Malabarba et al., 2020; Pompili et al., 2020; Schröpfer and Flachowsky, 2021).

This review aims to summarize recent achievements made to increase transformation efficiency and improve the precision of genetic modification in apples. It also summarizes the current state of methods available when genetically modified plants are used to improve breeding efficiency. Finally, it provides a brief overview of field trials with genetically modified apple plants and products available on the market that have been produced using such plants. In addition, shuttle systems other than *A. tumefaciens* for genetic modification of apple are briefly discussed.

## Methodological Approaches to Improving Transformation Efficiency

As in many other plant species, *A. tumefaciens*-mediated transformation is the most widely adopted procedure in *Malus* functional gene studies (Kost et al., 2015; Wurdig et al., 2015; Campa et al., 2019; Dalla Costa et al., 2020; Pompili et al., 2020; Ding et al., 2022). Although numerous publications on *in vitro* experiments/transformation in *Malus* (reviewed in Teixeira Da Silva et al., 2019 and Song et al., 2019) pertain to *Malus* × *domestica*—the domesticated apple, regeneration and transformation efficiency remains extremely low even for the apple, which is a model species in *Rosaceae* family of plants (Hanke et al., 2020). Thus, as successful genetic engineering relies on transformation efficiency, researchers have exploited several methods to increase efficiency.

Transformation efficiency in *Malus* appears to depend on the genotype as well as the regenerative ability of the explant used, of which age of explant, is also crucial. A few popular apple cultivars including “Gala” and its sport “Royal Gala,” “Jonagold,” “Elstar,” and “Pinova” as well as the rootstock cultivar M26 have been reported to produce high transformation efficiencies although the exact numbers are not reported. The definition of “high transformation efficiency” is subjective in literature and is perhaps misconstrued for “stable and efficient transformation.” It remains unclear for example, if efficiency should be calculated as the number of transformants emerging from a single leaf or several leaf explants of the same single leaf. Nonetheless, in our laboratory, we have also observed low degrees of transformation efficiency with apple cultivars, albeit stable. For example, a research publication originating from our laboratory (Wurdig et al., 2015) observed efficiencies between 0% and 0.4%. Recently, Wada et al. (2020) presented an apple rootstock Japan Morioka 2 (JM2) for stable and efficient transformation following several studies (Wada et al., 2009; Mimida et al., 2011; Tanaka et al., 2016; Yamane et al., 2019). However, experiments with transformation efficiencies reported were also low and ranged from 0.25% to 3.8% (Wada et al., 2020).

Leaf explants from fully expanded *in vitro* culture are the most commonly used in apple transformation experiments for their better regenerative ability and uniformity in growth (Magyar-Tabori et al., 2010). However, Magyar-Tabori et al. (2010) reinforced that regeneration of apple explants also depends largely on the genotype, and the interaction between genotype and cytokinins. Since regeneration and transformability in apple appear to be genotype-dependent, and factors other than *Agrobacterium* interaction and T-DNA transfer affect transformation efficiency (Maximova et al., 1998), we cannot rule out genetic factors. Tan et al. (2017) evaluated leaf regenerative heritability of 231 *Malus* genotypes and variations in bud regeneration rate (BRR) and the number of adventitious buds (NAB) formed per explant. The study of Tan et al. (2017), which included 78 hybrids of “Jonathan” × “Golden Delicious,” found that whereas BRR of “Jonathan” and “Golden Delicious” were 0% and 13.33%, respectively, a segregation ranging from 0% to 46.6% was observed, suggesting some hybrids were transgressive hybrids, with a high broad sense heritability (92.16%) for regeneration rate—a strong evidence that regeneration ability is heritable.

*In vitro* regeneration capacity in plant species has benefited from the breakthrough identification of the gene *BBM* in *Brassica napus*, which provided more insights into embryogenesis (Boutilier et al., 2002). The *BABY BOOM* gene, a member of the APETALA2/ETHYLENE RESPONSE FACTOR (AP2/EFR) family of transcription factors, was upregulated during *in vitro* induction of embryo in *B. napus* pollen grains and subsequent overexpression implicated it in cell proliferation and morphogenesis during embryogenesis (Boutilier et al., 2002). Subsequent studies in different species (Irikova et al., 2012; Florez et al., 2015; Horstman et al., 2017; Yavuz and Caliskan, 2021) confirmed the role of *BBM* in embryogenesis. *BBM* improves transformation including in otherwise recalcitrant species (Heidmann et al., 2011; Jha and Kumar, 2018; Nelson-Vasilchik et al., 2018). However, studies have observed pleiotropic phenotypes including excess shoot formation, which imply a role of *BBM* gene in somatic embryogenesis (SE; Horstman et al., 2017), and due to the localization of *BBM* in the cytoplasm instead of the nucleus following glucocorticoid receptor (GR)-mediated *BBM*-induction. The *BBM* gene activity is thereafter regulated by treatment with DEX—a strong synthetic glucocorticoid (Heidmann et al., 2011). The transformation efficiency of *BBM* was recently reported in apple—the first demonstration in *Malus* and in a fruit tree species (Chen et al., 2022). These authors identified two *Malus* × *domestica BBM* (*MdBBM*) genes designated as *MdBBM1* and *MdBBM2* in the apple genome. Overexpression of GR-mediated *MdBBM1* (MdBBM1-GR) resulted in medium shoot regeneration enhancement especially in two transgenic lines—MGB3 and MGB8, compared to control plants (Chen et al., 2022). The study of Chen et al. (2022) showed some interesting outcomes. Firstly, shoot enhancement was observed in transgenic lines without DEX treatment, and subsequent DEX treatment did not enhance shoot regeneration, suggesting that the *BBM* mediated by GR fusion was directly localized in the nucleus and not in the cytoplasm in apple, unlike in other species, e.g., pepper (Heidmann et al., 2011), where it had been previously reported that *BBM-GR* fusion causes a localization in the cytoplasm, which upon treatment with DEX subsequently leads to relocation into the nucleus. Secondly, *MdBBM1* alone without the GR fusion protein led to the regeneration of lines with two distinct shoot architecture-phenotypes not observed with *MdBBM1-GR* (Chen et al., 2022). Thirdly, *MdBBM1*-lines were shown to have enhanced cell division in leaf tissues. Perhaps most interesting in the study of Chen et al. (2022) is the high transformation efficiency of *MdBBM1* transformants used as explants in subsequent transformation experiments, which suggests that *BBM* is suitable in apple as a pre-transformation step, before subsequently introducing a target gene of interest. The use of *MdBBM1* transformants in *Agrobacterium*-mediated transformation experiments with a vector containing a mutant gene encoding acetolactate synthase (*ALS*), led to the generation of multiple transgenic shoots, with a transformation efficiency up to 31% (Chen et al., 2022). The *ALS* gene encodes for an enzyme that catalyzes the initial step of the biosynthetic pathway for essential branched-chain amino acids such as leucine, isoleucine, and valine (Mccourt and Duggleby, 2006). Herbicides like chlorsulfuron act as ALS inhibitors, thus affecting plant development leading to death (Tan et al., 2006). A dominant resistance to ALS inhibitors can be achieved by specific point mutations of the *ALS* gene, which was shown already for *M. × domestica* (Yao et al., 2013).

The use of *BBM1* in combination with the mutated version of *ALS* could be a game changer in apple transformation studies and should be replicated in other genotypes and wild species of *Malus*. In any case, an alternative selection marker instead of *nptII* is needed for a second transformation. Finding such a marker that works for different apple genotypes is not that easy. The removal of the selectable marker gene by combining *BBM*/*ALS* or *BBM*/*nptII* in a recombinase system could offer solutions to the problem.

Another innovative tool for gene delivery proven to increase transformation efficiency is by *Agrobacterium*-mediated transient expression of the genes *via* vacuum infiltration, also called agroinfiltration—a method first developed for plant-virus interaction (Grimsley et al., 1986). An agroinfiltration system developed by Lv et al. (2019) using four apple cultivars—“Red Fuji,” “Granny Smith,” “Royal Gala” and “Golden Delicious” reported the highest infiltration efficiency for the latter cultivar, which also supports genotype-dependent transformation efficiency. Transient silencing of the apple anthocyanin biosynthesis-gene, *MdMYB10*, proved the success of this method as a tool for functional gene analyses in apple as anthocyanin content in the cultivar “Red Fuji” was altered (Lv et al., 2019). Similarly, Chevreau et al. (2019) showed that *Agrobacterium*-mediated vacuum infiltration of “Gala” explants in inoculum containing Silwet surfactant proved significantly more efficient than wounding explants prior to submersion in bacterial suspension.

Notwithstanding all the progress that has been made in recent years in increasing transformation efficiency, great efforts are still needed to make genetic transformation an efficient tool for functional genomics in apple.

## Methods for Transient Expression of Gene Constructs

Methods for transient expression are especially valuable for organisms that are either not amenable for genetic transformation, or for species where the generation of transformants is a very lengthy process, such as perennial woody species like apple. Virus-induced gene silencing (VIGS) is one of such fast and efficient method that does not require stable transformation (Dommes et al., 2019). VIGS exploits existing plant defense mechanisms against viruses, where intruding viruses activate the RNA-mediated post-transcriptional gene silencing (PTGS) pathway (Baulcombe, 1999; Voinnet, 2001). Upon entry of a virus into a plant cell, its genome undergoes replication and double-stranded RNA arises (Rössner et al., 2022). Such double-stranded RNAs are processed into short-interfering RNAs (siRNAs). Most of the released siRNAs lead to PTGS *via* mRNA degradation, in a few cases *via* translational inhibition. Additionally, siRNAs spread systemically throughout the plant by cell-to-cell movement and *via* the phloem for long distances. As such, VIGS not only silences the gene of interest locally at infection sites but can reach most parts of the whole plant through systemic spreading.

The first step in VIGS is the successful delivery of the viral vectors into plant cells, for which several methods have been developed. The virus vectors can be delivered effectively by particle bombardment (Sasaki et al., 2011; Yamagishi and Yoshikawa, 2013; Werner Ribeiro et al., 2020). Here, just germinated apple seedlings are bombarded with gold particles loaded with viral RNA. The young seedlings can then be planted and silencing effects are generally observed after the second or fourth leaf (Yamagishi and Yoshikawa, 2013; Werner Ribeiro et al., 2020). For investigating the effect of gene silencing on the apple fruit, the fruit can be directly injected with *A. tumefaciens* infiltration solution, that contains the VIGS vectors between its T-DNA left and right border sequences (Li et al., 2016; Jiang et al., 2017; Ding et al., 2022). The bacterial solution can also be used for virus transmission by submerging whole plantlets (Zhang et al., 2016).

For successful gene silencing *via* the VIGS system, both organisms need to interact well to allow each of the molecular steps required for PTGS and systemic spread to function. Therefore, it is plausible that specific viruses can only infect a specific set of plant species. Two viruses have been successfully used for VIGS in apple: *Tobacco rattle virus* (*TRV*) and the *Apple latent spherical virus* (*ALSV*; Ratcliff et al., 2001; Sasaki et al., 2011; Yamagishi and Yoshikawa, 2013; Zhang et al., 2016; Dommes et al., 2019; Werner Ribeiro et al., 2020).

The *ALSV* was first isolated from an apple tree (Li et al., 2000) and has been shown to be an efficient VIGS vector for several plant species (Li et al., 2004; Igarashi et al., 2009), including apple, pear, and Japanese pear (Sasaki et al., 2011). For most plant species the infection with *ALSV* is without disease symptoms, which allows proper characterization of plant phenotypes after gene silencing. The virus RNA can easily be propagated on *Chenopodium quinoa* plants (Yamagishi and Yoshikawa, 2013; Werner Ribeiro et al., 2020). The *ALSV* genome consists of two positive single-stranded RNAs, each harboring a single open reading frame (Li et al., 2000; Gedling et al., 2018; Werner Ribeiro et al., 2020). RNA1 contains all proteins that are essential for genome replication, and RNA2 encodes movement and capsid proteins (Gedling et al., 2018; Werner Ribeiro et al., 2020). These sequences have been modified to serve as viral vectors. For both vectors, pEALSR1, and pEALSR2L5R5, a 35S promoter, and a nopaline synthase terminator have been added. Additionally, a multiple cloning site has been added to pEALSR2L5R5, which allows easy insertion of 200–400 bp fragments of the gene of interest (Li et al., 2004; Werner Ribeiro et al., 2020). In cases, where *A. tumefaciens* is used for transmission of viruses, the left and right border sequences need to be added to the vector. This virus was first tested in apple with constructs silencing the photosynthesis genes *alpha subunit of chloroplast chaperonin 60a and ribulose-1, 5-bisphosphate carboxylase small subunit*, the housekeeping genes *elongation factor 1 alpha (EF-1a)* and *actin*, and the flowering time gene *MdTFL1* (Sasaki et al., 2011). In addition, the two squalene synthase isoforms *MdSQS1* and *MdSQS2* were successfully silenced in apple tree using *ALSV* as a vector for VIGS (Navarro Gallón et al., 2017), as well as *MdERF2* (Li et al., 2016).

Like *ALSV*, *TRV* is also a positive single-stranded virus that consists of two vectors (Ratcliff et al., 2001). It was successfully used for VIGS in crabapple to downregulate *McMYB10*, a regulatory gene of anthocyanin production (Zhang et al., 2016). Further, *TRV* was used successfully to silence a diverse set of genes in apple: *MdHB1* or *MdMYB10* (Jiang et al., 2017), *MdbZIP2*, *MdbZIP39*, *MdbZIP80*, *MdIPT5b* (Feng et al., 2021), *MdCNGC2* (Zhou et al., 2020), and *MdAP2_1a* (Ding et al., 2022). The HD-Zip I transcription factor MdHB1 and the MYB transcription factor MdMYB10 are negative and positive regulators of anthocyanin biosynthesis, respectively (Jiang et al., 2017). Also the AP2/EREBP transcription factor MdAP2_1a plays a role in anthocyanin pigmentation (Ding et al., 2022). MdIPT5b is a key enzyme of cytokinin biosynthesis and appears to be regulated by the bZIP transcription factors MdbZIP2, MdbZIP39, MdbZIP80, that form repressive heterodimers (Feng et al., 2021). MdCNGC2 is a cyclic nucleotide-gated ion channel, which appears to be a negative regulator of resistance to the fungal pathogen *Botryosphaeria dothidea* (Zhou et al., 2020). *Botryosphaeria dothidea* is the causal agent of ring rot, which leads to fruit rot and also death of apple trees. A stronger immune response was found in *MdCNGC2*-silenced plants (Zhou et al., 2020).

Since silencing *via* siRNAs bears the disadvantage of occasional off-target silencing, VIGS-vectors have also been designed with artificial miRNA sequences that replace the short target gene sequence -MIR-VIGS (Tang et al., 2010). Thus, the transmitted miRNA rather than siRNAs will be responsible for gene silencing. Although to our knowledge MIR-VIGS has not been applied in apple yet, artificial miRNAs have been successfully used for stable gene silencing in apple, using a miR156h backbone (Charrier et al., 2019b).

In addition to systemic silencing, VIGS-vectors can also be used for overexpression of a gene of interest—virus-induced overexpression (VOX). The functionality of this idea was demonstrated by Li et al. (2004), who were able to show through GFP expression that it is possible to express foreign genes in apple using artificially generated virus vectors. Later, this ALSV system was used, for example, by Yamagishi et al. (2011) to transiently express the flowering integrator gene *FT* in apple seedlings. To achieve stable expression of the desired protein in plant cells, the sequence of an autoproteolytic peptide has to be added to the vector, so that the protein of interest can be released (Rössner et al., 2022). This was successfully done with the apple virus *ALSV* for using it as a VOX vector in quinoa roots (Ogata et al., 2021). This vector system however only allows small inserts due to the small packaging size, which does not allow the overexpression of large genes.

VIGS has also been adopted to support plant defense against pathogens—host-induced gene silencing (HIGS; Rössner et al., 2022). Here, the sequence that triggers silencing is not a plant sequence, but one that comes from specific pathogens. This however has not been applied to apples yet.

Apart from silencing and overexpression, VIGS has also been adapted to genome editing—virus-induced genome editing (VIGE). The TRV viral system has been adapted and used to introduce parts of the CRISPR-Cas system into plants and has been successfully used for genome editing in several agronomically important monocot and dicot plant species (Rössner et al., 2022). As the vectors TRV is also suitable for apple infections, this protocol could easily be adapted for apple.

## Genome Editing

Genome editing (GE) covers several technical approaches that allow precise genetic modifications such as insertions and/or deletions (indels) or base substitutions at desired target loci in living organisms including humans, plants, and microbes (Manghwar et al., 2019). GE technologies have emerged as powerful tools for crop breeding as well as for basic plant research (Hua et al., 2019). For all GE applications, the precise induction of DNA breaks in the genome of the organism to be modified is an underlying principle. This is achievable today with various artificial endonucleases. Different types of artificial nuclease systems have been used for GE in plants during the last two decades, including, zinc finger nucleases (ZFNs), transcriptional activator-like effector nucleases (TALENs), and the clustered regularly interspaced short palindromic repeat (CRISPR)/CRISPR-associated nuclease (Cas) system (Puchta and Fauser, 2014). These nuclease systems can be engineered to recognize and bind specific sequences at the desired target locus. Acting as endonucleases, DNA breaks are introduced at this site, serving as initiator signal for various GE events (Schmidt et al., 2019).

**Figure 2:**
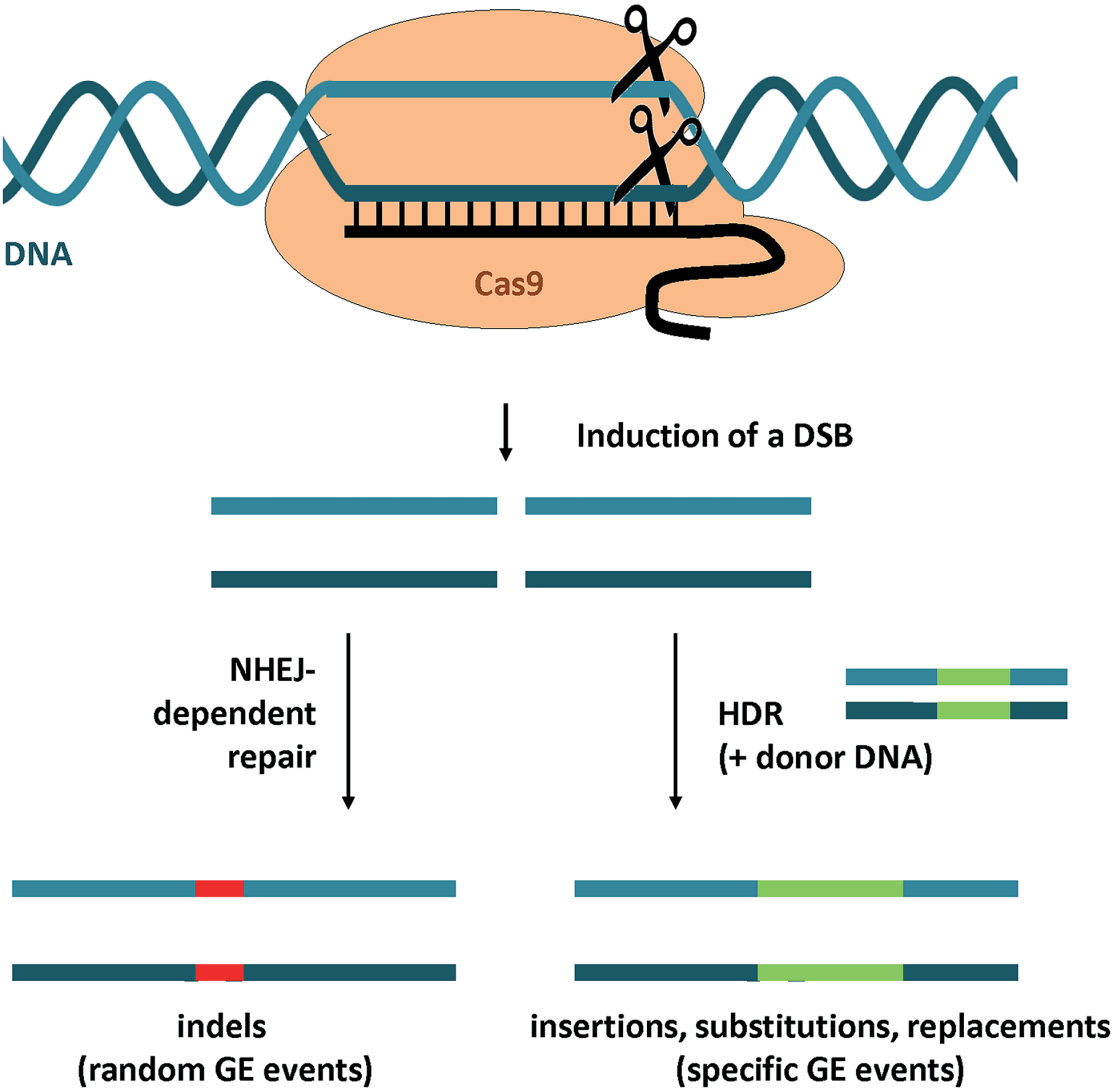
GE strategies using CRISPR/Cas9. A DSB in the target locus is introduced by CRISPR/Cas9-mediated DNA cleavage. The DSB repair by NHEJ leads to random mutations at the site of the DNA break including insertions and deletions (indels). By the addition of a homologous donor DNA, the DSB can be repaired by HDR. This strategy can be used to introduce specific GE events such as insertions, substitutions or gene replacements.

For the repair of double-stranded DNA breaks (DSBs), plants possess two major pathways (Figure 2) that differ in mechanism, repair fidelity, and frequency (Schröpfer et al., 2014). The highly efficient non-homologous end-joining (NHEJ) pathway is the predominant repair pathway in plants and can be described mechanistically simplified as a simple re-ligation of the broken and processed DSB ends. NHEJ is associated with random mutations at the target site such as small insertions and/or deletions (indels). Therefore NHEJ-dependent GE approaches are favored for the creation of knock-out and loss-of-function mutations. Alternatively, the DSB can be repaired by homology-directed repair (HDR), which is dependent on a homologous sequence serving as a DNA template during the repair. By adding additional donor DNA template with sequence homology to the predicted DSB site, targeted sequence insertions or gene replacements can be executed by HDR (Manghwar et al., 2019). HDR allows precise control of GE events, but the efficiency is low compared to NHEJ (Puchta, 2005).

The first report on GE on apple was published in 2015, using a heat-shock inducible ZFN for targeted mutagenesis of an artificial *GUS* reporter gene in transgenic apple lines (Peer et al., 2015). However, due to its simplicity and flexibility, the CRISPR/Cas system has emerged as the most successful and user-friendly tool for GE applications in the past 10 years.

## Genome Editing With Crispr/Cas9

Described as a programmable RNA-guided DNA endonuclease and discovered as a part of the adaptive bacterial immunity (Jinek et al., 2012), the CRISPR/Cas9 system originating from *Streptococcus pyogenes* was the first to be used for GE (Jinek et al., 2012; Feng et al., 2013). The effector nuclease Cas9 forms a complex with a guide (g)RNA, that exhibits sequence homology to the target DNA. Cas9 recognizes protospacer adjacent motifs (PAM) in the DNA and stays at sites that are complementary to the gRNA sequence. Subsequently, structural changes in the complex lead to the activation of the nuclease activity, which results in the formation of a DSB (Jiang and Doudna, 2017). The CRISPR/Cas9 system is the best-known CRISPR system and has been the most widely used over the past 10 years. The first application of CRISPR/Cas9 in apple was described in 2016, which was used for the targeted mutagenesis of the endogenous reporter gene *MdPDS*, coding for a phytoene desaturase that is essential for chlorophyll production (Nishitani et al., 2016). Subsequent reports on GE in apple have been based mainly on the use of CRISPR/Cas9 to date (Table 1). However, the variety of available CRISPR systems that differ in their properties for GE applications is growing steadily. During evolution, CRISPR/Cas systems have undergone diversification, and two classes and six types have been discovered that differ in the composition of Cas effector proteins, the processing of the pre-CRISPR (cr)RNA, and the diversity of domains in the nuclease protein (Koonin et al., 2017). Class 1 CRISPR/Cas systems include types I, III, and IV and require multiple subunits of the Cas effector. Only one single effector nuclease is involved in class 2 systems (types II, V, and VI; Makarova et al., 2020), which makes them attractive for GE applications. Types II and V are suitable for DNA editing, while type VI is employed for RNA editing (Moon et al., 2019). Cas9 belongs to type II systems and orthologs from several species such as *S. pyogenes* (*Sp*Cas9), *Staphylococcus aureus* (*Sa*Cas9), and *Campylobacter jejuni* (*Cj*Cas9) differing in PAM sequence, protein size, spacer length, and other genome editing properties were already used for GE (Moon et al., 2019). Furthermore, engineered Cas9 variants are available such as xCas9, which can recognize a broader range of PAMs, has more editing specificity, higher efficiency, and lower off-target effects than *Sp*Cas9 (Manghwar et al., 2019). Cas9 needs a second trans-acting crRNA (tracrRNA) for gRNA processing and cleavage (Stephenson et al., 2018). The tracrRNA and pre-crRNA form a hybrid molecule by hybridization whereby the tracrRNA acts as a scaffold in the forming ribonucleotide protein (RNP) complex. For most GE applications, a chimeric single guide (sg)RNA of about 100 nt is used, consisting of both RNAs.

**Table 1:** Overview of published reports on CRISPR/Cas-mediated GE of apple.

CRISPR-tool	Target organism(s)	Target gene(s)	Strategy	Construct design	CRISPR/Cas delivery	Detection of GE events	Resulting edited tissue	GE events	References
*Sp*Cas9; *Lb*Cas12a	*Malus domestica*	*MdPDS* (different cultivars)	Pre-selection of efficient gRNAs by *in vitro* cleavage assays	Pre-assembled CRISPR RNP complexes	-	-	-	-	Schröpfer et al., 2021
*Sp*Cas9	*Malus sieversii* (wild apple)	*MsPDS*	Gene knock-out using two sgRNAs	pYL-CRISPR/Cas9: PUBI-Cas9, AtU3d-P-sgRNA, HygR; pTG-CRISPR/Cas9: P35S-Cas9, AtU6-26-sgRNA(2x), KanR	Stable T-DNA transformation (*Agrobacterium tumefaciens*)	Albino phenotype (*MsPDS* knock-out); Sanger sequencing of cloned products	*In vitro* calli; one chimeric mutated bud	Small deletions (1–28 bp); small insertions (1–2 bp)	Zhang et al., 2021b
*Lb*Cas12a	*M. domestica* (“Gala”)	*MdPDS*	Multiplex targeted mutations with two gRNAs; tracing GE events in chimeric regenerates	*At*Ubq10-P-*Lb*Cas12a,*At*U6-P-sgRNAs (2x); KanR	Stable T-DNA transformation (*A. tumefaciens*)	Albino phenotype (*MdPDS* knock-out); fluorescent PCR capillary gel electrophoresis; amplicon deep sequencing; Sanger sequencing of cloned products	*In vitro* shoots; chimeric and homohistont; heterozygous and biallelic mutations; genotypically uniform mutant with biallelic mutations at two target loci	Small deletions (Ø12-13 bp); no large deletions between neighbored target sites;	Schröpfer and Flachowsky, 2021
*Sp*Cas9	*M. domestica* (and pear)	*MdPDS*	Dechimerization by adding an adventitious regeneration step from leaves of the primary transgenic plants (T0)	pDE-Cas9: *Pc*Ubi4-2-P-*Sp*Cas9, *Md*U3-sgRNA1, *Md*U6-sgRNA2, KanR	Stable T-DNA transformation (*A. tumefaciens*)	Albino phenotype (*MdPDS* knock-out); Sanger sequencing of cloned products	*In vitro* shoots; complex pattern of editing in T0 shoots; elimination of wild allele in regenerated tissue; chimeric and biallelic	Deletions, substitutions, insertions	Malabarba et al., 2020
*Sp*nCas9-cytidine base editor (CBE)	*M. domestica* (and pear)	*MdALS & MdPDS* (“Gala”)	Multiplex base editing of two different reporter genes (*Md*ALS and *Md*PDS) by two guide RNAs	*Pc*Ubi4-3-P*-Sp*nCas9-PmCDA1-UGI, MdU3-sgRNA1, MdU6-sgRNA2, KanR	Stable T-DNA transformation (*A. tumefaciens*)	Albino phenotype (*MdPDS* knock-out); growth on chlorsulfuron selection medium (*MdALS* knock-out); Sanger sequencing of cloned products	*In vitro* shoots	C-to-T DNA substitutions; other substitutions, deletions	Malabarba et al., 2020
*Sp*Cas9	*M. domestica* (and grape vine)	*MdDIPM4*, *MdDIPM4* & *MdDIPM1*	(1) on-target GE and (2) T-DNA excision from genome by heat-shock inducible Flp/FRT system	*At*Ubq10-P-*Sp*Cas9, *At*U6-P-sgRNAs (1-2x),Hsp17.5-E-P-Flp,KanR; flanked by FRT-sites	Stable T-DNA transformation (*A. tumefaciens*)	Amplicon deep sequencing	Regenerated tissue	(1) Deletion (1–7 bp) in target genes and (2) not reported	Dalla Costa et al., 2020
Cas9	*M. domestica*	*MdCNGC2*	Knock-out of gene function to improve resistance to *Botryosphaeria dothidea*; use of two gRNAs	35S-P-Cas9, MdU6-P-sgRNA1, UBQ10-P-sgRNA2, KanR	Stable T-DNA transformation (*A. tumefaciens*)	Sanger sequencing of cloned products	*In vitro* callus	Small deletions and insertions at only one target locus; chimerism and status of mutations not reported	Zhou et al., 2020
*Sp*Cas9	*M. domestica* (“Gala,” “Golden Delicious”)	*MdDIPM4*	(1) Knock-out of gene function to reduce susceptibility to *Erwinia amylovora* and (2) T-DNA excision from genome by heat-shock inducible Flp/FRT system	*At*Ubq10-P-*Sp*Cas9, *At*U6-P-sgRNA,Hsp17.5-E-P-Flp,KanR; flanked by FRT-sites	Stable T-DNA transformation (*A. tumefaciens*)	Amplicon deep sequencing	Mutated apple plant lines	(1) Mostly small deletions (1–27 bp), small insertions (1 bp); substitution and (2) T-DNA excision reported	Pompili et al., 2020
*Sp*Cas9	*M. domestica* (“Gala”; and pear)	*MdPDS*, *MdTFL1.1*	Multiplex GE with two gRNAs per target gene; generation of T-DNA-free edited lines	*Pc*Ubi4-2-P-*Sp*Cas9, *Md*U3-sgRNA1, *Md*U6-sgRNA2, KanR	Stable and transient T-DNA transformation (*A. tumefaciens*)	Albino phenotype (*MdPDS* knock-out); early flowering (*TFL1.1* knock-out); Sanger sequencing of cloned products	*In vitro* shoots, chimeric tissue, complex editing profiles, biallelic mutations; T-DNA-free edited *in vitro* shoots	Deletions (1–29 bp), insertions (+1 bp), substitutions, inversion, duplication	Charrier et al., 2019a
*Sp*Cas9	*M. domestica* (and grape vine)	Not specified	Proposal of protocols for: (1) plasmid-mediated GE and (2) DNA-free GE in protoplasts	SpCas9-GFP; KanR	(1) Stable T-DNA transformation (*A. tumefaciens*) and (2) direct delivery of CRISPR–Cas9 RNPs in protoplasts	PCR-RFLP, Cel-1 assay, heteroduplex mobility analysis (HMA)	-	-	Osakabe et al., 2018
*Sp*Cas9	*M. domestica* (and grape vine)	*Md*DIPM1, *Md*DIPM2, and *Md*DIPM4	(1) Determination of *in vitro* cleavage efficiency of several gRNAs and (2) DNA-free GE in protoplasts	Pre-assembled CRISPR RNP complexes	Direct delivery of CRISPR RNPs in protoplasts	Amplicon deep sequencing	Protoplasts	Deletions, insertions	Malnoy et al., 2016
*Sp*Cas9	*M. domestica*	*MdPDS*	Knock-out of gene function using single gRNAs	35S-P-SpCas9-GFBSD2, AtU6-1-P-sgRNA	Stable T-DNA transformation (*A. tumefaciens*)	Albino phenotype (*MdPDS* knock-out); Sanger sequencing (direct or cloned PCR products)	*In vitro* shoots, chimeric, biallelic	Deletions (1–8 bp), insertions (1 bp)	Nishitani et al., 2016

## Genome Editing With Crispr/Cas12a

Cas12a, which was previously named Cpf1 (CRISPR from *Prevotella* and *Francisella 1*; Zetsche et al., 2015), is the best-known representative of the type V CRISPR nucleases. Only recently, the first application of Cas12a in apple was reported (Schröpfer and Flachowsky, 2021). Different orthologs of Cas12a were isolated from *Francisella novicida* U112 (*Fn*Cas12a), *Acidaminococcus* spp. BV3L6 (AsCas12a), and *Lachnospiraceae bacterium* ND2006 (*Lb*Cas12a) and assessed for GE in eukaryotes (Safari et al., 2019; Alok et al., 2020). For target recognition, Cas12a prefers a T-rich PAM sequence (5′-TTTV-3′; V is A, G, or C; Zetsche et al., 2015), which is different from Cas9 which uses a G-rich PAM (Anders et al., 2014). These distinct properties expand the possible spectrum of target sequences since additional AT-rich sequences that are characteristic, e.g., for plant promotor regions or introns can be targeted by Cas12a (Safari et al., 2019; Wolter and Puchta, 2019). Furthermore, Cas9 and Cas12a differ in cleavage position relative to the PAM and the produced cleavage products. The cut introduced by Cas9 is located proximal to the PAM and produces blunt ends or ends with 1 nt overhangs (Jinek et al., 2012; Zuo and Liu, 2016). In contrast, the Cas12a cleavage site is located distal to the PAM and its nucleolytic activity leads to staggered ends with 5–8 nt long 5′-overhangs (Zetsche et al., 2015). The cleavage position of Cas12a, located far away from the PAM and seed region, was postulated to promote site-directed integration events (Zetsche et al., 2015). Therefore, the use of Cas12a is advantageous for the targeted integration of DNA by *in planta* gene targeting (ipGT) and HDR-based applications, which was already demonstrated in *A. thaliana* (Wolter and Puchta, 2019). Cas12a does not require a second tracrRNA molecule and the mature crRNA of Cas12a is much shorter compared to Cas9. The length of the crRNAs of Cas12a is 42–44 nt and includes a 23–25 nt-long target-specific guide sequence (Zetsche et al., 2015). The shortness of the crRNA allows a cost and time-efficient generation of crRNAs by chemical syntheses, which is advantageous for applications with pre-assembled RNPs, e.g., for pre-selection of efficient crRNAs by *in vitro* assays on target sequences (Schröpfer et al., 2021). In addition, the smaller molecular size of Cas12a represents a further benefit for DNA-free GE by the application of RNPs *in vivo* in future. However, some limitations of Cas12a such as their temperature sensitivity have to be considered for further GE applications on plants. Cas12a exhibits a reduced enzymatic activity at ambient temperatures, which is mandatory for plant transformation and cultivation (Moreno-Mateos et al., 2017; Bernabe-Orts et al., 2019; Lee et al., 2019; Malzahn et al., 2019; Schindele and Puchta, 2020). Thus, the efficiency of GE applications using Cas12a can be further enhanced by, e.g., selecting temperature-tolerant Cas12a variants, such as the optimized tt*Lb*Cas12a (Schindele and Puchta, 2020) or the use of newly identified Cas12a orthologs with high-level editing activity (Zhang et al., 2021a).

## CRISPR/Cas-Dependent Base Editors

**Figure 3:**
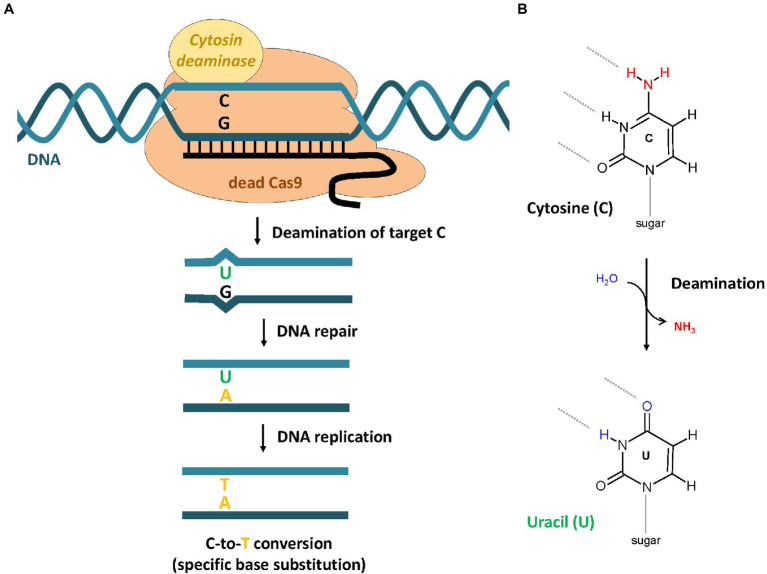
Base editing by CRISPR-dependent cytosine base editor (CBE). **(A)** The CBE binds specifically to the target DNA, which is defined by the guide RNA sequence. **(B)** The fused cytosine deaminase catalyzes the deamination of the cytosine base, which results in the formation of uracil. Because of the changed base-pairing properties, the G-U pair forms a mismatch that is recognized by the DNA repair machinery of the cell. Repair events can result in the replacement of the original G to A. The C-to-T conversion becomes stable after replication.

Mutagenesis of target genes by NHEJ-dependent GE results in random mutations, which cannot be controlled directly by the CRISPR/Cas system. In contrast, HDR-dependent GE using a repair template allows the accurate determination of the desired mutation, but the method is very inefficient in plants. A new CRISPR-mediated technology called base editing (BE) was developed and enables precise nucleotide substitutions (C-to-T or A-to-G) without requiring a donor template and which is independent of the introduction of DSBs (Komor et al., 2016; Mishra et al., 2020). It is based on the use of base editors, which consists of a catalytically inactive Cas9 variant [dead-Cas9 (dCas9) or Cas9 nickase (nCas9)] and a cytosine or adenosine deaminase domain. The cytosine base editor (CBE) removes an amino group from cytosine which leads to the formation of uracil (Figure 3). The resulting U-G mismatch is repaired by the DNA repair machinery of the cell to form a U-A base pair. During the following replication, T gets in incorporated into the newly synthesized, and the C-to-T conversion is manifested. To avoid frequent removal of uracil by the uracil DNA glycosylase (UDG), second-generation base editors (BE2) contain an additional uracil DNA glycosylase inhibitor (UGI; Komor et al., 2016). The major improvement of third-generation base editors (BE3) was the replacement of dCas9 with nCas9, which introduce a single-stranded DNA break in the opposite strand of the deaminated cytosine (Mishra et al., 2020). An adenine base editor (ABE) base-editing strategy results in A-to-G conversion, which is initiated by the deamination of adenine resulting in the formation of inosine (Gaudelli et al., 2017; Figure 3). Base editors have already been successfully applied to several model and crop plant species such as Arabidopsis, rice, tomato, wheat, and maize (Shimatani et al., 2017; Zong et al., 2017; Hua et al., 2018; Kang et al., 2018). Recently, the first study on the use of CBE on apple was published (Malabarba et al., 2020). In this study, a CBE system including a Cas9 nickase, *Pm*CDA1, and a UGI was used successfully to induce C-to-T substitutions in the reporter genes *MdALS* and *MdPDS*.

## Progress in Genome Editing of Apple

Since 2016, a series of studies on GE of apple have been reported, aiming for the targeted mutation of endogenous target genes (Table 1). To date, *Agrobacterium*-mediated transformation has played an indispensable role in the delivery of the CRISPR/Cas tool in apple. Most of the GE studies on apple are based on the use of the CRISPR/Cas9 system from *S. pyogenes*. Very recently, the application of further CRISPR tools such as CBEs or CRISPR/Cas12a was established for apple (Malabarba et al., 2020; Schröpfer and Flachowsky, 2021). For the establishment of GE techniques, the targeted mutagenesis of the endogenous reporter genes such as the phytoene desaturase gene (*MdPDS*) or the acetolactate synthase (*MdALS*) is of great use due to the easy scoring of mutant phenotypes. The knock-out of the single-copy gene *PDS*, which is essentially involved in chlorophyll biosynthesis (Qin et al., 2007), results in albino phenotype in *M. ×domestica* and the wild apple species *Malus sieversii* (Nishitani et al., 2016; Charrier et al., 2019a; Malabarba et al., 2020; Schröpfer and Flachowsky, 2021; Zhang et al., 2021b).

Further studies were published aiming for the modification of traits by targeted mutagenesis of apple genes using the CRISPR/Cas9 system to increase disease resistance or to induce early flowering. In this context, the knock-out of *MdDIPM* genes (susceptibility-associated gene DspA/E interacting protein), which were expected to mediate susceptibility to the fire blight causing agent *Erwinia amylovora*, was the focus of several studies (Malnoy et al., 2016; Dalla Costa et al., 2020; Pompili et al., 2020). It was reported that genome-edited apple lines exhibiting mutations in *MdDIPM4* showed less fire blight symptoms (reduced on average to 50%) after infection with *E. amylovora* (Pompili et al., 2020) compared to control plants. With the aim to improve resistance to ring rot caused by the infection with *B. dothidea*, *MdCNGC2* (cyclic nucleotide-gated ion channel 2) was targeted by CRISPR/Cas9 and mutated *in vitro* callus material was investigated (Zhou et al., 2020). Further, CRISPR/Cas9-mediated indel mutations in the gene *MdTFL1.1* (terminal flower 1.1), which was already known as a floral repressor in apple, resulted in an early flowering phenotype of edited *in vitro* shoots (Charrier et al., 2019a).

## Rapid Cycle Breeding

In addition to the use of genetic modification methods for the direct improvement of traits in established cultivars, there have been repeated attempts to use genetically modified plants to increase efficiency in breeding. Breeding of apple is a lengthy process, which takes 15–25 years before a new cultivar can be released to the market (Kellerhals and Meyer, 1994; Wannemuehler et al., 2019). This is mainly due to the long duration of the generation period, which is mainly determined by the duration of the juvenile phase of cross-bred progeny. This juvenile phase lasts 5–10 or even 12 years (Visser, 1964; Fischer, 1994). Targeted shortening of the juvenile phase could reduce the duration of breeding programs by several years. This would simplify otherwise complex programs, such as introgression of traits from genetically distant wild relatives, which often require several generations of crossing, as well as drastically reduce the length of time required. Shortening the juvenile phase in apple is possible by overexpression of flowering genes or by silencing of repressor genes that suppress flowering genes during the juvenile phase. This has been demonstrated with stably transformed plants (Kotoda et al., 2003; Flachowsky et al., 2007, 2012; Trankner et al., 2010; Tanaka et al., 2014), but also with transient expression of gene constructs using a virus vector system (Hanke and Flachowsky, 2010; Yamagishi et al., 2016).

**Figure 4:**
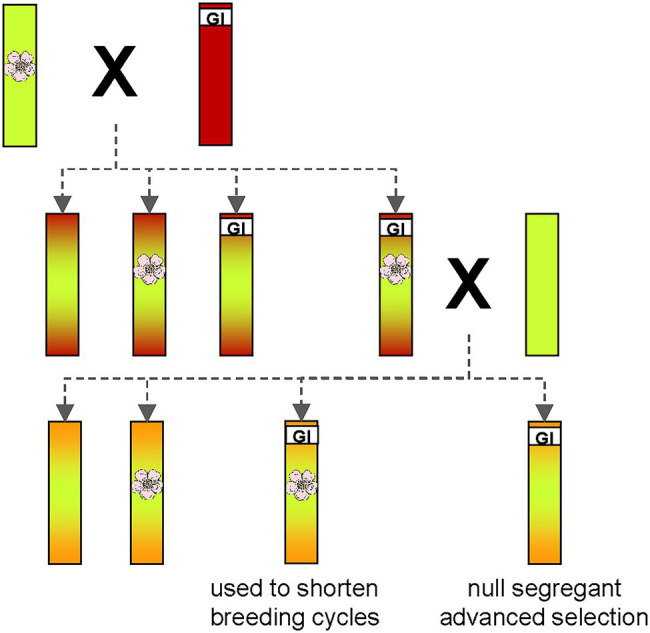
Schematic representation of the idea behind the rapid cycle breeding program. One parent with excellent traits in terms of fruit quality (green) containing a transgenic T-DNA insertion for early flowering is crossed with another parent (red) containing a trait of interest, e.g., disease resistance. Many resistances are unfortunately only found in *Malus* wild species, which usually have insufficient fruit quality. The high proportion of adverse alleles in these genotypes is symbolized by the red color. Repeated backcrossing with high-quality apple varieties (green) gradually reduces the proportion of negative alleles. The flower symbolizes the transgene for early flowering. GI is the abbreviation for “gene of interest,” which confers the desired trait as, e.g., disease resistance. If both traits (early flowering and resistance) are inherited monogenically, a quarter of offspring will be produced with both traits. These genotypes can then be used in further crosses to shorten breeding cycles. Another quarter of offspring will contain only the gene of interest. Such non-transgenic null-segregants can be released from the breeding program as advanced selections.

Using such an early flowering transgenic apple plant, Flachowsky et al. (2009) established the concept of rapid cycle breeding (Figure 4).

The idea was to use an early-flowering transgenic plant as a parent in crosses to transfer the early-flowering trait to some of the progeny. If this transgenic parent contains a single locus T-DNA insertion in its genome, the transgenic insertion will be inherited in half of the offspring. If this parent is crossed with another parent that has a monogenic trait of interest (e.g., disease resistance), about a quarter of the resulting offspring will contain both, the early flowering gene and the gene conferring the trait of interest. Such progeny is then selected for further crossing steps. In each following generation, a quarter of offspring containing both traits is produced. Subsequently, another quarter of offspring contains only the gene of interest. Such plants, called non-transgenic null segregants, are selected from the last generation of the breeding program and represent advanced selections that are free of unwanted transgenic DNA. Producing such an advanced selection is also possible using traditional breeding, but this would take a much longer period.

In proof-of-concept experiments, Flachowsky et al. used the transgenic early flowering apple clone T1190 (Flachowsky et al., 2009, 2011), which was derived from the transformation of PinS, an offspring from a cross between “Pinova” and “Idared” (Luo et al., 2019), with the *BpMADS4* gene of birch (Flachowsky et al., 2007). T1190 was used as it contains a single copy of the transgenic insertion on chromosome 4 (Flachowsky et al., 2011; Luo et al., 2019) and is characterized by a short juvenile phase and a growth habit that results in a sufficient number of fruits and seeds if used in crosses. T1190 was used as maternal parent and crossed with the wild apple genotype *Malus fusca* (Raf.) C.K.Schneid. MAL0045 (Emeriewen et al., 2020) to introduce resistance to *E. amylovora* (Burr. Winslow et al. 1920), which causes fire blight disease. Transgenic fire blight resistant progeny were then used in crosses to introduce further resistances (e.g., apple scab, powdery mildew, fire blight resistance from other sources). Using T1190, a generation time of less than 1 year could be achieved (Flachowsky et al., 2011).

In parallel, T1190 was also used in a Swiss apple breeding program for the introgression of fire blight resistance. Le Roux et al. (2012) crossed T1190 with the fire blight-resistant ornamental apple “Evereste.” Transgenic F1 seedlings carrying the 273 bp allele of the SSR (Simple Sequence Repeat) marker ChFbE06, which co-segregates with the fire blight resistance locus Fb_E (Parravicini et al., 2011), were selected and used in crosses with other elite apple cultivars. To identify fire blight-resistant BC'1 (pseudo-backcross generation 1) seedlings with the lowest possible “Evereste” genome content, Le Roux et al. (2012) performed a background selection using SSR markers evenly distributed across the apple genome. This allowed the identification of two BC'1 seedlings carrying less than 15% of the genome of “Evereste.” In the following years, Schlathölter et al. (2018) were able to use the material generated by Le Roux et al. (2012), to produce seven advanced BC'3 and 11 BC'4 selections. Schlathölter et al. (2018) demonstrated impressively that with this rapid cycle apple breeding system, five cross-bred generations are possible in only 7 years.

Although this system could speed up apple breeding enormously and the goals formulated by the European Green Deal could be achieved much sooner, it is still unclear whether null-segregants of the rapid cycle breeding approach may be used in practical breeding. It is still not clear whether or not they fall under the existing regulations for genetically modified organisms. Critics of this technology fear that there could be further, yet undetected genetic modifications in the genome of these plants. However, Patocchi et al. (2021) allayed those fears by demonstrating that in T1190, there are at least no additional and unexpected transgenic insertions.

**Figure 5:**
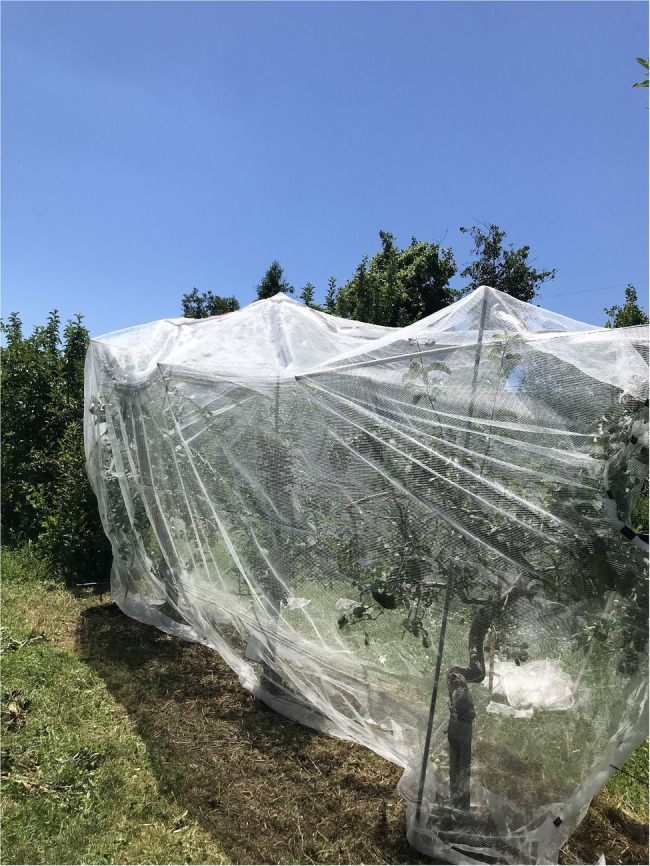
Netted *BpMADS4* transgenic apple trees used for crosses in the experimental orchard at Cornell University. Nets around the trees prevent pollinators from spreading the transgenic pollen outside the orchard. Source: Dr. Awais Khan who leads the rapid cycle breeding program at Cornell University in Geneva (New York, United States) kindly provided the photograph.

In contrast, the situation in the United States is completely different. There, null-segregants obtained by the rapid cycle breeding approach can be freely planted in the open field without any restrictions (Callahan et al., 2016). Transgenic plants used for crosses can also be planted in the field, as long as they are netted (Figure 5).

This makes this system interesting for United States apple breeders, which is also reflected in the fact that some have already started to use it. Within the RosBREED project,^4^ T1190 was used to pyramid and combine resistances against fire blight and apple scab (Iezzoni, 2019), for example. In 2020, the US Department of Agriculture’s National Institute of Food and Agriculture awarded a new grant to Cornell AgriTech researchers to combat fire blight (Cornell Chronicle, 2020) and to continue the work that has been started with the rapid cycle breeding system. In another project, T1190 was used to start the introgression of blue mold resistance from *M. sieversii* wild apples into apple breeding material (Luo et al., 2020).

It cannot be ruled out that the products of United States apple breeding will reach the European market one day. For this reason, European apple breeders are afraid of a competitive disadvantage if these genotypes are not deregulated in the EU (Laurens et al., 2018).

## Field Trials and Genetically Modified Apple Products

A complete overview of the field trials with genetically modified (gm) apples carried out worldwide does not exist. Several authors have tried to compile such an overview for apples (e.g., Hanke et al., 2020), but also for other plants (e.g., Slot et al., 2018). However, the internet addresses for the authorities of different countries mentioned in these publications have changed or are no longer active. Comprehensive information on field trials with gm apples is available for Europe and the United States. No records were found for Australia (AGDH, 2022), Japan (Food Safety Commission of Japan, 2022), Canada (CFIA, 2022), and Brazil (CTNBio, 2022). Flachowsky and Hanke (2011) published a summary of the number of field trials in the United States and Europe some time ago. In the United States, 106 field trials have been notified or applications for deliberate release have been documented in the Animal and Plant Health Inspection Service (APHIS) database of the United States Department of Agriculture (USDA) since 1991. Eight were denied, 20 were withdrawn, but 78 were issued or acknowledged, depending on whether it was a notification or a request for approval (permit). It is interesting to note that the number of applications has been quite constant over the last 30 years with 31 between 1991 and 2000, 30 between 2001 and 2010, and 40 between 2011 and 2020. For 2021, five applications are documented. In the last 10 years, there even seems to be a slight increase. The traits that had been genetically modified in the plants intended for release were as follows: improved fruit quality, altered ripening, and storability, reduced browning of the fruit flesh due to reduced polyphenol oxidase activity, increased cold tolerance, accelerated flowering, and altered flower morphology (Hanke et al., 2020). In contrast, the EU GMO register lists only eight summary notifications (SNIFs) and only two of them were published after 2011.^5^ The last three field trials with genetically modified apples in the EU ended between 2019 and 2021. One field trial, which was carried out in Sweden (notification number: B/SE/14/13820) with gm rootstocks of M9 and M26 expressing the *rolB* gene of *Agrobacterium rhizogenes*, ended in December 2019. Another field trial (notification number: B-NL-15-L01) was carried out in the Netherlands. This trial, where cisgenic apples transformed with the R6 allele of the *MdMYB10* promoter was evaluated, ended in January 2020.

The last field trial (notification number: B/NL/10/05), which was also carried out in the Netherlands ended in April 2021. This field trial was focused on the evaluation of intra- and cisgenic apple plants of “Gala” expressing the *HcfVF2* (*Rvi6*) scab resistance gene of *M. floribunda* 821. Outside the EU, there was a field trial with cisgenic plants in Switzerland. Apple plants transformed with the *FB_Mr5* gene for fire blight resistance of *M.* ×*robusta 5* were subjected to a multi-level assessment (Schlathölter et al., 2021). This trial is also already finished. The fact that in the meantime there are no more gm apple trials in the EU is among other things also due to the existing GMO regulations and the lack of acceptance in parts of the population.

Despite all the developments, there is so far only one commercial product in the world made from genetically modified apples. The Okanagan Specialty Fruits™ (OSF) has introduced non-browning apple varieties of the Arctic® series into the market. In these apples, the activity of a polyphenol oxidase (PPO) was reduced by RNAi-mediated gene silencing (Stowe and Dhingra, 2021). The Arctic® Granny and Arctic® Golden varieties have been deemed “as safe and nutritious as traditional apple varieties” by the United States Department of Agriculture (USDA), the Food and Drug Administration (FDA), the Canadian Food Inspection Agency (CFIA) and Health Canada. They can be grown, sold, and consumed in the United States and Canada (Hanke et al., 2020).

## Limitations and Alternatives to *Agrobacterium*-Mediated Transformation

*Agrobacterium*-mediated transformation is an established method for gene functional studies and breeding research in apples, with a wide range of applications. However, there are limitations associated with this method that are technical and societal, and consequently political. The technical limitations lie mainly in the limited tendency of the apple to regenerate and the associated comparatively low transformation efficiency. Thus, the transformation of apple remains very time- and labor-intense, and success is not always certain. This scares off many research groups from investing in this technology. On the social level, the presence of transgenic DNA sequences in the plants produced, as well as other changes in the genome, which are often not clearly defined by critics, are discussed as possible risks. Especially, the presence of transgenic sequences in the genome of the target organism leads to a strong rejection of this technology and the resulting GMOs in parts of society. This is one of the reasons why gm approaches have not played a significant role in breeding new cultivars for fruit production worldwide. Only one breeding program by the biotech company Okanagan Specialty Fruits has succeeded in commercializing non-browning gm apple varieties to date, known as Arctic® apples, in Canada and the United States (Stowe and Dhingra, 2021).

In recent years, the availability of new, high-precision breeding technologies, such as CRISPR/Cas-mediated GE, are generating great interest, which avoid the integration of foreign DNA or which are completely independent of DNA. Especially for vegetatively propagated crops like apple, which is self-incompatible and characterized by a heterozygous genome, the elimination of transgenic sequences by outcrossing is not meaningful. Outcrossing can take decades and can lead to unintentional loss of the edited trait (e.g., traits that are due to a mutation) and cultivar-specific features of the edited genotype will be lost in the offspring. To avoid a stable integration of the T-DNA, transient *Agrobacterium*-mediated transformation experiments with time-limited herbicide selection were performed. Using this strategy, Charrier et al. (2019a) were able to identify single T-DNA-free, genome-edited apple lines. However, transient *Agrobacterium*-mediated transformation carries the risk of unwanted incorporation of the delivered genetic sequences into the plant genome, which can lead to off-target effects and uncertainty in the regulation of the resulting edited plants. Other studies aimed to minimize the trace of exogenous DNA by inducing an FLP/recombination system, which was successfully demonstrated for apple (Pompili et al., 2020). Using this strategy, the majority of transgenic sequences can be removed from the genome after the establishment of a GE line. However, small stretches of foreign DNA remain in the region of the T-DNA border sequences after T-DNA excision, which is permanently preserved in the genome as a footprint of the former T-DNA insertion. Consequently, the resulting plant is a GMO according to the current EU directives. Furthermore, this technology is hampered by a high effort for the characterization of the T-DNA integration events and the resulting lines (Dalla Costa et al., 2020). The first study on DNA-free GE of apple was published by Malnoy et al. (2016), using pre-assembled CRISPR/Cas9 RNPs for PEG-mediated delivery into apple protoplasts. Genome-edited protoplasts could be identified in this study, suggesting this method will pave the way for the generation of DNA-free GE apple plants. However, the regeneration of intact plants from apple protoplasts is still a challenge. To the best of our knowledge, an efficient and reproducible protocol for the regeneration of intact plants from apple protoplasts is currently not available. There are only a few reports in which intact apple plants could be regenerated from protoplasts (Patatochatt et al., 1988, 1993; Wallin and Johansson, 1990; Perales and Schieder, 1993). Whether this strategy will be pursued further, due to the low chance of success, remains to be seen.

Thus, the delivery of the site-specific nucleases in the form of proteins considerably minimizes the risks of off-target effects and eliminates uncertainty regarding DNA-based transformation methods. In this context, alternative delivery methods based on cell-penetrating peptides (CPPs) or nanoparticles are attractive and will be developed in future for plants. In contrast to the mammalian system, where these vector systems have already been successfully established, e.g., for the internalization of drugs, the cell wall of plant cells is an additional barrier and represents a huge challenge to be overcome (Wang et al., 2021). CPPs, also known as protein translocation domains (PTDs), membrane translocating sequences, or Trojan peptides, are small peptides of 6 to 30 amino acids residues that can be used as a non-viral and non-bacterial cargo carrier system for delivery of large molecules into living cells (Huang et al., 2015). CPPs can penetrate and translocate across the plasma membrane, either alone or carrying various types of biomolecules such as nucleic acids (DNA, RNA) or proteins. The categorization of CPPs is according to their origin (protein-derived peptides, model peptides, designed peptides) or their chemical properties (amphipathic, non-amphipathic; Soliman et al., 2022). A prominent member of protein-derived CPPs is the Tat peptide, which was the first CPP identified and shown to mediate cell penetration and nuclear localization of the trans-activator of transcription (TAT) protein of human immunodeficiency virus 1 (HIV-1; Vives et al., 1997). Other examples for CPPs are TP10, M918, pVEC, or R9. CPPs can differ in various characteristics such as cell and species specificity, but they have to be proven to be universal carriers for all eukaryotic cells (Soliman et al., 2022). The CPP-mediated delivery of DNA, RNA, and proteins into plant cells and tissue was already demonstrated by several studies, which are reviewed by Soliman et al. (2022). Furthermore, using organelle-specific CPPs, distinct cellular compartments can be addressed, including the nucleus, mitochondria, and plastids/chloroplasts. The successful application of CPPs for non-transgenic GE *in planta* has been demonstrated in proof-of-concept studies, using CPPs D-R9 and (BP100)2K8 for the delivery of ZFNs proteins in wheat microspores and haploid embryos (Bilichak et al., 2015; Ng et al., 2016; Soliman et al., 2022). An extension of this CPP-mediated transfer of other GE systems such as CRISPR/Cas RNPs or even further proteins or nucleic acids applications is well conceivable and the subject of current research projects.

In addition, nanoparticles (NP) are considered as potential delivery vectors for plant systems. Three groups of nanoparticles are defined: organic (lipids, proteins, or polymers); hybrid (nanofoams); and inorganic (metals or salts), and different forms are available, including polymeric nanoconstructs, nanomembranes, nanotubes, nanofibers and nanosized silicon drips (Silva et al., 2019). Until now, only a few studies on the use of nanoparticles for plants have been reported, which are summarized by Wang et al. (2021).

Independent of the delivery of GE tools, a particular focus in GE of apple is the non-intentional generation of chimeric plants. By tracing GE events during the regeneration of apple shoots it has already been shown that multiple GE events in different cell lines lead to a chimeric composition of the GE tissue (Schröpfer and Flachowsky, 2021). This problem was already addressed previously by the work of Malabarba et al. (2020), who demonstrated that multiple rounds of regeneration lead to efficient dechimerization. Particularly in light of these facts, the use of informative and sensitive methods for the analysis of the target loci in GE tissue is necessary. For this purpose, methods such as amplicon deep sequencing or fluorescent PCR capillary gel electrophoresis are suitable (Schröpfer and Flachowsky, 2021). A further challenge for the application of new breeding technologies to apple is explained by the complexity of the apple genome characterized by a high degree of heterozygosity and the presence of multiple gene copies and large gene families. Therefore, for the modification of breeding relevant traits, simultaneous targeting of multiple genomic loci will be necessary. Various studies have already demonstrated that multiplex GE is achievable by the use of distinct guide RNAs (Table 1) and the generation of non-chimeric edited tissue with biallelic mutations of multiple loci is feasible (Schröpfer and Flachowsky, 2021). However, a major limitation for breeding applications is the availability of suitable target genes whose function is already well characterized. Here, *Agrobacterium*-mediated transformation in combination with the new GE techniques offers an excellent opportunity to analyze the function of specific candidate genes in more detail by targeted mutagenesis. However, whether the resulting plants can then be evaluated sufficiently and under natural conditions in the field depends on the applicable political regulatory framework.

## Conclusions AND Future Research

Great efforts have been made over the last 30 years to establish *Agrobacterium*-mediated transformation in apple. Numerous groundbreaking successes have been achieved, such as the establishment of protocols for the production of marker gene-free gm plants, the production of cisgenic apple plants or the targeted mutagenesis of gene loci by means of GE. Methods have been developed that can make breeding more efficient, such as rapid crop cycle breeding, and numerous field trials with gm apples have been carried out in many countries around the world. In the end, however, only one product, the non-browning Arctic® apples, has been successfully launched on the market so far.

Nevertheless, the *Agrobacterium*-mediated transformation method has become an indispensable method in the field of breeding research. The function of numerous genes has been investigated by overexpression or RNAi-based gene silencing. In addition, genes have already been characterized with the help of GE in initial studies. Progress has also been made in the field of transient expression of genes or gene constructs. Despite all this progress, the future success of this technology will be limited primarily by its efficiency. Increasing transformation efficiency should therefore remain one of the research goals for the future.

## Author Contributions

All authors listed have made a substantial, direct, and intellectual contribution to the work and approved it for publication.

## Conflict of Interest

The authors declare that the research was conducted in the absence of any commercial or financial relationships that could be construed as a potential conflict of interest.

## Publisher’s Note

All claims expressed in this article are solely those of the authors and do not necessarily represent those of their affiliated organizations, or those of the publisher, the editors and the reviewers. Any product that may be evaluated in this article, or claim that may be made by its manufacturer, is not guaranteed or endorsed by the publisher.

## Footnotes

^1^ https://www.fao.org

^2^ www.prognosfruit.eu

^3^ https://www.rosaceae.org

^4^ https://www.rosbreed.org/

^5^ https://webgate.ec.europa.eu/fip/GMO_Registers/
